# Western diet-induced ultrastructural changes in mouse pancreatic acinar cells

**DOI:** 10.3389/fcell.2024.1380564

**Published:** 2024-03-14

**Authors:** Saška Lipovšek, Jurij Dolenšek, Barbara Dariš, Ismael Valladolid-Acebes, Tanja Vajs, Gerd Leitinger, Andraž Stožer, Maša Skelin Klemen

**Affiliations:** ^1^ Faculty of Medicine, University of Maribor, Maribor, Slovenia; ^2^ Department of Biology, Faculty of Natural Sciences and Mathematics, University of Maribor, Maribor, Slovenia; ^3^ Gottfried Schatz Research Center, Division of Cell Biology, Histology and Embryology, Medical University of Graz, Graz, Austria; ^4^ Faculty of Chemistry and Chemical Engineering, University of Maribor, Maribor, Slovenia; ^5^ The Rolf Luft Research Center for Diabetes and Endocrinology, Karolinska Institutet, Karolinska University Hospital, Stockholm, Sweden

**Keywords:** acinar cells, autophagy, lipid droplets, mitochondria, necrotic cells, rough endoplasmic reticulum, western diet

## Abstract

Mouse models of diet-induced type 2 diabetes mellitus provide powerful tools for studying the structural and physiological changes that are related to the disease progression. In this study, diabetic-like glucose dysregulation was induced in mice by feeding them a western diet, and light and transmission electron microscopy were used to study the ultrastructural changes in the pancreatic acinar cells. Acinar necrosis and vacuolization of the cytoplasm were the most prominent features. Furthermore, we observed intracellular and extracellular accumulation of lipid compounds in the form of lipid droplets, structural enlargement of the cisternae of the rough endoplasmic reticulum (RER), and altered mitochondrial morphology, with mitochondria lacking the typical organization of the inner membrane. Last, autophagic structures, i.e., autophagosomes, autolysosomes, and residual bodies, were abundant within the acinar cells of western diet-fed mice, and the autolysosomes contained lipids and material of varying electron density. While diets inducing obesity and type 2 diabetes are clearly associated with structural changes and dysfunction of the endocrine pancreas, we here demonstrate the strong effect of dietary intervention on the structure of acinar cells in the exocrine part of the organ before detectable changes in plasma amylase activity, which may help us better understand the development of non-alcoholic fatty pancreas disease and its association with endo- and exocrine dysfunction.

## Introduction

The pancreas is composed of an exocrine and an endocrine part. Acinar cells of the exocrine part synthesize and secrete digestive enzymes that are collected by the pancreatic ductal system and funneled into the duodenum to aid in food assimilation. Pancreatic endocrine cells, on the other hand, synthesize and secrete into the bloodstream at least 5 types of hormones that are involved in the whole-body homeostasis of glucose and other energy-rich nutrients (Danielsson et al., 2014). If the endocrine function is no longer sufficient to meet an increased demand for insulin due to decreased insulin sensitivity in target tissues (the liver, adipose tissue, and skeletal muscle), type 2 diabetes mellitus (T2DM) develops (Turner et al., 1979; Khan et al., 2003).

The pancreas in mice is not as well defined as in humans; it is diffusely distributed in a dendritic manner and composed of a duodenal, splenic, and gastric lobe (Liu et al., 2010; Dolenšek et al., 2015). Underneath the fibrous capsule, the connective tissue extends into the parenchyma and divides the tissue into larger lobes and smaller lobules (Liu et al., 2010; In’t Veld and Marichal, 2010). Each lobule consists of acini formed by pyramid-shaped acinar cells (Motta et al., 1997). The exocrine cells comprise about 96%–99% and the endocrine cells about 1%–4% of the total pancreas parenchyma (Saito et al., 1978; Rahier et al., 1981).

The acinar cells of the exocrine pancreas are crucial for synthesis of digestive enzymes, while pancreatic ductal cells secrete a bicarbonate-rich fluid that neutralizes the acidic gastric juice in the duodenal lumen, thus providing a suitable environment for the action of secreted enzymes (Petersen and Tepikin, 2008; Hegyi et al., 2011). The digestive enzymes in zymogene granules are stored in an inactive proenzyme form and are released from the cells via exocytosis in response to stimulation with neurohormonal agents, including acetylcholine, cholecystokinin, gastrin-releasing peptide, substance P, vasoactive intestinal peptide (VIP), and secretin. The zymogen granules are secreted into the acinar lumen, from which they pass the ductal tree to be finally released into the duodenum, where activation of the enzymes occurs (Argent et al., 2012).

T2DM, one of the greatest threats to global public health in the western world, is associated with obesity, insulin resistance, and, eventually, with reduced insulin secretion (Della Corte et al., 2015). Insulin resistance in key target organs and beta cell dysfunction are the two key factors driving T2DM. In addition, the development of T2DM is at least partly linked to excessive fat accumulation in the liver and the pancreas. The phenomenon where increased fat accumulation occurs in these organs without excessive alcohol consumption is termed non-alcoholic fatty liver disease (NAFLD) and non-alcoholic fatty pancreas disease (NAFPD) (Della Corte et al., 2015). The latter leads to both endocrine and exocrine dysfunction, resulting in glucose plasma dysregulation, reduced exocrine pancreatic function, and aggravation of acute pancreatitis. Importantly, it also seems to increase the susceptibility to pancreatic cancer (Hardt et al., 2000; Alempijevic et al., 2017; Taylor et al., 2018; Overton and Mastracci, 2022).

The development of T2DM is therefore the result of a complex interaction between multiple genetic and environmental factors. A promising way to better understand the mechanisms of T2DM development is to use mouse models of T2DM. The most frequently used genetic models, i.e., the *ob/ob* and *db/db* mice, are genetically determined and do not reflect the degree of heterogeneity seen in human T2DM (Srinivasan and Ramarao, 2007; Wang et al., 2014). In contrast to genetic models, diet-induced models take advantage of the fact that predisposed mice develop obesity and diabetes when they are fed specific diets (Srinivasan and Ramarao, 2007). It is known that the diet-induced models better reflect the multifactorial human nature of disease than the genetic models (King, 2012).

In 1988, Surwit and co-workers demonstrated for the first time that diet-induced obesity (DIO) impairs glucose metabolism in C57BL/6J mice (Surwit et al., 1988). Feeding mice a high-fat diet (HFD) with 58%–60% of calories from fat, 20%–36% from simple carbohydrates and 4%–20% from protein for 11–20 weeks immediately after weaning resulted in obesity, hyperglycemia and hyperinsulinemia (Surwit et al., 1988), impaired glucose tolerance (Gallou‐Kabani et al., 2007), an increase in the fat depots with the highest gain in the mesenteric fat pads (Rebuffe-Scrive et al., 1993; Parekh et al., 1998), hypercholesterolemia (Rebuffe-Scrive et al., 1993), and an increase in liver triglycerides (Kobayashi et al., 2004). Compared to other strains, C57BL/6J mice were shown to be predisposed to respond to the dietary intervention only after developing visceral obesity (Rebuffe-Scrive et al., 1993), which resembles T2DM in humans. However, there are also several drawbacks to the HFD model. Mice exposed to HFD immediately after weaning respond to increased metabolic demand with beta cell hyperplasia and increased beta cell mass, which robustly compensates for the increased peripheral insulin resistance, in contrast to human T2DM etiopathogenesis, where in a large proportion of patients, beta cell adaptation and compensation are only transient (Tschen et al., 2009; Stožer et al., 2019; Klemen et al., 2024). Furthermore, the proportion of fat in various HFDs (58%–60%) is high compared to the percentage in the modern human western diet (WD), which is estimated at about 30% (National Research Council, 2005; Lai et al., 2014).

To increase the translational relevance, in our study we used a WD to induce obesity and diabetes in adult C57Bl/6J mice, i.e., not immediately after weaning. A WD in humans, also called a standard American diet, is characterized by an increased intake of food with high fat and simple sugar content, along with excessive consumption of red meat, refined cereals, and high-fat milk products (Odermatt, 2011). The WD used in our current study containing 40% of calories from fat, 43% from simple carbohydrates, and 17% from protein is more similar in composition to the western human diet than HFD (Valladolid-Acebes et al., 2015).

We have recently demonstrated that analyzing the ultrastructural properties of pancreatic cells using TEM can be a useful tool to detect early morphological changes related to changes in pancreas physiology (Klemen et al., 2022). It is known that in NAFPD and T2DM, fat accumulation occurs not only in the islet cells but also intralobularly in pancreatic acinar cells as well as interlobularly (Korc et al., 1981; Williams and Goldfine, 1985); however, to the best of our knowledge, a detailed qualitative analysis of ultrastructural changes related to diet-induced T2DM has not yet been performed. Therefore, we set out to systematically analyze the ultrastructural changes in acinar cells of WD-fed mice and found necrosis, vacuolization and autophagy, as well as an impaired ultrastructure of the RER and mitochondria.

## Material and methods

### Ethical statement

The protocol of this study was approved by the Administration for Food Safety, Veterinary Sector and Plant Protection, Ministry of Agriculture, Forestry and Food, Republic of Slovenia (approval number U34401-12/2015/3) and Swedish ethics committee (approval number 6362-2023). The study was conducted in strict accordance with all national and European recommendations pertaining to care and work with laboratory animals. The authors complied with the ARRIVE guidelines.

### Animals, diets, and experimental setups

The study was carried out on 12-week-old male mice C57BL/6J (RRID: IMSR_JAX:000664) purchased from Charles River. The mice were fed with a standard rodent diet (CD, R70, Lantmännen, Stockholm, Sweden) containing 72% kcal from carbohydrates, 10% kcal from fat, and 18% kcal from protein until 12 weeks of age. From 12 to 20 weeks of age, the mice were divided into 2 groups. In the control group (CD), the mice continued to be fed with the standard rodent diet (*n* = 11), while in the WD-fed group, the mice were fed a western diet (*n* = 11) containing 43% kcal from carbohydrates, 40% kcal from fat and 17% kcal from proteins (D12079B, Research diets inc., New Jersey, United States). Nutritional profile of the diet is presented in Table 1. During the experimentation, food and water were available *ad libitum*. Mice were euthanized before isolation and dissection of the pancreas for the light and transmission electron microscopy analysis. Five WD-fed and five CD-fed mice were used for the characterization of the animal model. Another set of six WD-fed and six CD-fed mice were used for microscopy studies.

**TABLE 1:** Nutritional profile of the diet.

Diet	CD	WD
Nutritional profile		
Proteins, %	14.5	20
Nonessential amino acids	8.0	12.8
Essential amino acids	5.5	7.3
Fat, %	4.5	21.0
Cholesterol	0.018	0.21
Linoleic acid, %	1.00	5.39
Linolenic acid, %	0.19	1.48
Arachidonic acid, %	0.2	0.0
Saturated fatty acid, %	22.0	25.8
Monounsaturated fatty acids, %	23.0	12.6
Polyunsaturated fatty acids, %	53.80	2.86
Fiber, %	4.9	0.2
Carbohydrates, %	60.1	50.0
Minerals, %	2.65	0.04
Vitamins, %	<5.00	0.01
Water, %	<11	<9
Energy profile		
Energy, kcal/g	3.0	4.7
Proteins, % of kcal	17.7	17.0
Fat, % of kcal	10.5	40.0
Carbohydrates, % of kcal	71.7	43.0

### Intraperitoneal glucose tolerance test (ipGTT)

Glucose tolerance was monitored at the end of the studies in mice fasted for 6 h during daytime. Blood glucose concentrations were measured at basal state (0 min) and 15, 30, 60, 90, and 120 min after an intraperitoneal (ip) glucose injection (2 g/kg of body weight). Glucose concentrations were measured with an Accu-Chek Aviva monitoring system (F. Hoffmann–La Roche). IpGTTs were performed before assigning the animals to the different experimental groups and after 8 weeks of diet intervention, in the set of mice used for the characterization of the animal model.

### Intraperitoneal insulin tolerance test (ipITT)

IpITTs were performed in 6-h fasted mice, at the end of the studies, to determine whole-body insulin sensitivity. Blood glucose concentration was measured at basal state (0 min) and 15, 30, 60, 90, and 120 min after an ip injection of insulin [0.25 IU/kg of body weight, diluted in phosphate-buffered saline (PBS), Novo Nordisk]. Blood glucose concentrations were measured with an Accu-Chek Aviva monitoring system (F. Hoffmann–La Roche). IpITTs were performed in the set of mice used for the characterization of the animal model.

### Measurements of plasma insulin

At the end of the experiments blood samples were collected in Microvette CB 300 K2 EDTA tubes (SARSTEDT AG & Co. KG) in non-fasting conditions and after 6 h fasting (time point 0 of the ipITT). Thereafter, samples were centrifuged at 2,500 × *g* for 15 min at 4°C. Plasma was collected from the supernatant and preserved at −80°C until use. Insulin was analyzed using an ultrasensitive mouse insulin enzyme-linked immunosorbent assay (ELISA) kits (Crystal Chem Inc.), according to manufacturer’s instructions.

### Amylase activity and protein levels in pancreas and plasma

Blood samples and whole pancreases were obtained from non-fasted mice at the end of the diet intervention. Pancreas tissues were rinsed in phosphate saline buffer (PBS, Gibco) to remove excess blood and weighted before homogenization. Tissues were finely minced and homogenized in 20% (weight/volume) in amylase assay buffer (Merck) with a glass homogenizer on ice. For full tissue disruption, homogenates were next feezed with liquid nitrogen and thawed at room temperature three times. Homogenized pancreases were stored at −150°C until use. Blood was collected in Microvette CB 300 K2 EDTA tubes (SARSTEDT AG & Co. KG), and kept on ice. Thereafter, blood samples were centrifuged at 2,500 g for 15 min at 4°C; plasma was collected and preserved at −80°C until use. Amylase activity was measured, according to manufacturer’s instructions, in pancreas homogenates and plasma samples from CD- and WD-fed mice using an enzymatic method suitable for the colorimetric detection of amylase activity (Merck). Plasma and pancreas amylase concentration was measured, according to manufacturer’s instructions, by using a mouse-specific ELISA kit (LSBio). Total protein content was determined in both plasma and pancreas homogenates by using the Bradford’s method and the activity of the enzyme as well as its protein concentrations were corrected by total amount of proteins present in the samples analyzed.

### Light microscopy and transmission electron microscopy

Six WD-fed and six CD-fed mice were used for light microscopy (LM) and transmission electron microscopy (TEM). For LM and TEM, part of the splenic lobe of pancreas was cut into small pieces. From each individual, nine pieces of the tissue (measuring 1–8 mm^3^) were fixed in 2.45% glutaraldehyde and 2.45% paraformaldehyde in a 0.1 M sodium cacodylate buffer (pH 7.4) at room temperature for 3 h and at 4°C for 14 h, washed in a 0.1 M sodium cacodylate buffer (pH 7.4) at room temperature for 3 h and postfixed with 2% OsO4 at room temperature for 2 h. The tissue was dehydrated in a graded series of ethanol (50%, 70%, 90%, 96%, and 100%, each for 30 min at room temperature) and embedded in TAAB epoxy resin (Agar Scientific Ltd., Essex, England). From each piece of the tissue both thin and ultrathin sections were prepared. For light microscopy, semi-thin sections (500 nm) of the pancreas were stained with 0.5% toluidine blue in aqueous solution and analyzed by a Nikon Eclipse E800 light microscope equipped with the Nikon DN100 camera. Ultra-thin sections (75 nm) were cut with a Leica EM UC7 RT ultramicrotome and transferred onto copper grids, stained with uranyl acetate and lead citrate and analyzed by a Zeiss EM 900 transmission electron microscope. Complete ultrathin sections (the whole surface of the ultrathin section) of the tissue were observed at various magnifications (starting with 3,000 × magnification). In each ultrathin section we randomly selected and analyzed >100 acinar cells in detail. Structures were defined according to their ultrastructural characteristics. Nucleus was recognized by heterochromatin, euchromatin, outer and inner nuclear membrane, separating the content of the nucleus (nucleoplasm) from the rest of the cell. Mitochondria were recognized by their matrix, outer and inner membrane forming cristae. Rough endoplasmic reticulum (RER) was detected based on the presence of the network of flattened cisternae and presence of ribosomes on the outer membrane. Autophagosomes were selected as structures with a double layer membrane, containing electron-lucent material. When the outer membrane of an autophagosome fuses with a lysosome an autolysosome is formed and the inner membrane of the autophagosome degrades. The latter is thus a structure with a single layer membrane, containing electron-dense material. Vacuoles were recognized as structures lined with a single layer membrane (Motta et al., 1997; Nakatogawa, 2020).

### Morphometric analyses: quantification of structural changes on TEM images

To estimate and quantify the influence of WD on the shape of RER, RER was detected on TEM images using the “Trainable Weka Segmentation” plug-in in Fiji software employing machine-learning and image analysis (Arganda-Carreras et al., 2017). In brief, TEM images were cropped to contain RER-only segments. We first built a classifier by depicting RER structures; the classifier was then applied to individual images resulting in segmentation of RER cisternae. Data were expressed as percentage of cross-sectional area populated by RER cisternae on each individual TEM image. Mitochondria were manually outlined on TEM images, and cross-sectional (Equation 1) area and circularity parameter were calculated in Fiji software. The latter is defined as:

$$Circularity=\frac{4∙\pi ∙area}{{perimeter}^{2}}$$ (1)

The circularity equals 1 for a perfect circle and values close to 1 indicate a tendency toward a more circular shape.

## Results

### *In vivo* animal model characterization

**FIGURE 1:**
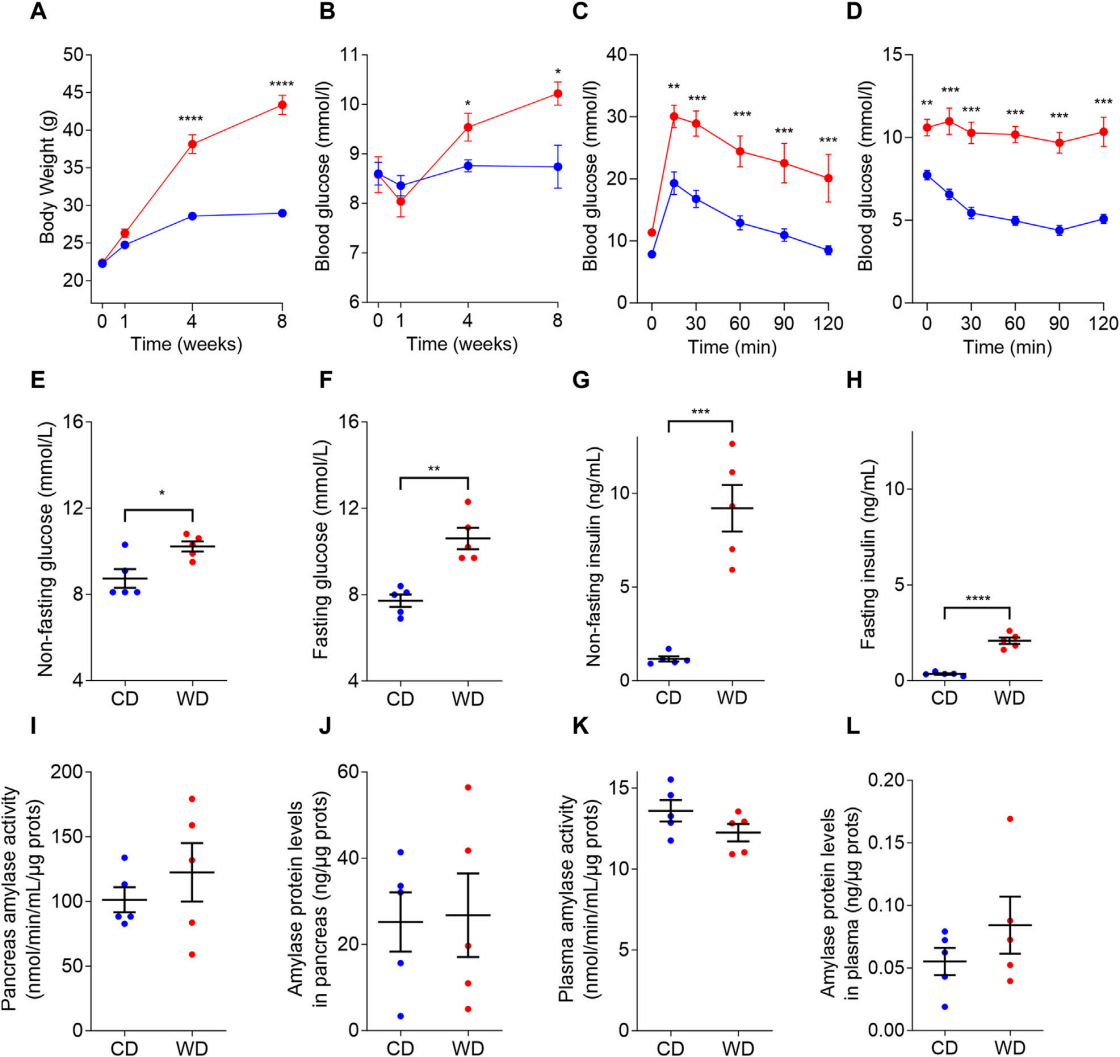
Diabetic-like glucose dysregulation induced in male C57BL/6J mice by western diet (WD, red color) compared to control diet (CD, blue color). **(A)** WD feeding induced increased body weight compared to CD. **(B)** Non-fasting plasma glucose levels in mice fed with CD and WD. **(C,D)** ipGTT **(C)** and ipITT **(D)** following 8 weeks of diet intervention. **(E–H)** Non-fasting **(E)** and fasting **(F)** blood glucose concentrations, non-fasting **(G)** and fasting **(H)** plasma insulin concentrations measured after 8 weeks of diet intervention. **(I–L)** Amylase activity in pancreas **(J)** and plasma **(K)**, and amylase protein levels in pancreas (L) and plasma (M). In all panels CD is depicted with blue and WD with red color. Data pooled from 5 mice per group and expressed as mean and SEM. Individual data points are additionally presented in panels **(E–L)**. *p < 0.05; **p < 0.01; ***p < 0.001; and ****p < 0,0001 (Student’s *t*-test for panels **(A,B,E–L)**; and 2-ANOVA followed by Bonferroni’s *post hoc* test for panels **(C–D)**. Otherwise, differences are non-significant (*p* > 0.05).

To induce diabetes-like glucose dysregulation in male C57BL/6J mice, we resorted to diet-induced obesity by feeding the animals with WD (Figure 1). The diet intervention resulted in obesity and non-fasting hyperglycemia already after 4 weeks of diet, culminating in a 50% increase in body weight (Figure 1A) and 1.5 mM higher non-fasting blood glucose levels after 8 weeks of diet intervention (Figure 1B). From the glycemic control standpoint, mice randomly assigned to the different groups did not show differences in their glucose tolerance and insulin sensitivity before the WD was given (Supplementary Figure S1). However, after 8 weeks of diet intervention, WD-fed animals displayed impaired glucose tolerance (Figure 1C) and elevated fed and fasting blood glucose levels, as compared to the CD-fed animals (Figures 1E, F). WD-induced hyperglycemia was accompanied by elevated plasma insulin concentrations, both in fed and in fasting conditions (Figures 1G, H). An ipITT performed at the end of the studies showed that after 8 weeks WD-fed mice were unable to respond to exogenously administered insulin and, thus, had insulin resistance as compared to the insulin-sensitive CD-fed mice (Figure 1D). We did not detect any changes in amylase activity and amylase protein levels in samples obtained from the pancreas or blood plasma (Figures 1I–L).

### Light microscopy

**FIGURE 2:**
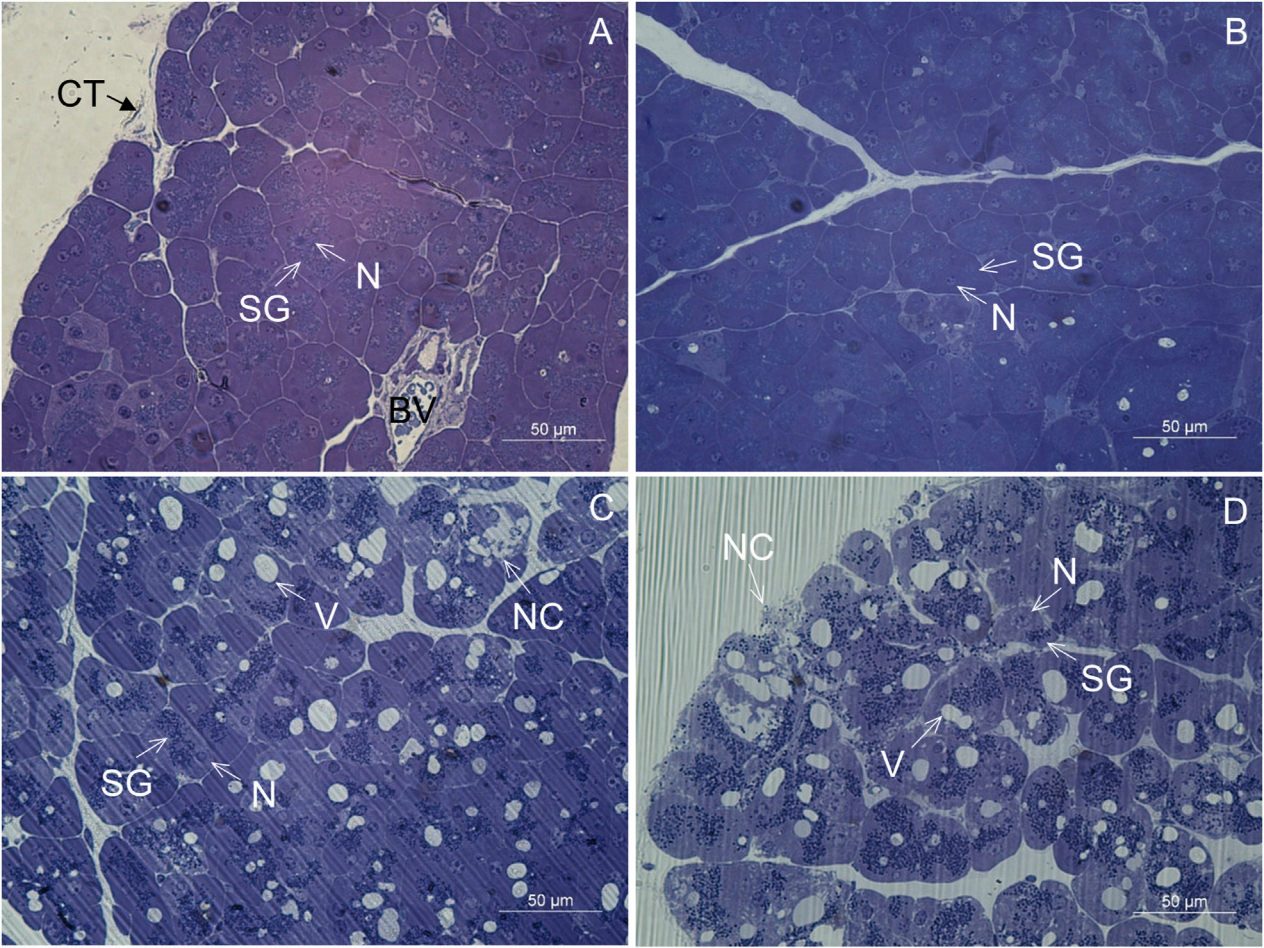
Semithin section of the pancreas. **(A,B)** Control mice. **(C,D)** Mice fed a Western diet. BV, blood vessel; CT, connective tissue; N, nucleus; NC, necrotic cell; SG, secretory granules; V, vacuole.

In CD-fed mice, the exocrine pancreatic acinus was composed of several acinar cells containing numerous secretory granules. The nucleus was round and located centrally or basally (Figures 2A, B). The exocrine pancreas had a rich capillary network (Figure 2A). On the periphery, a thin capsule of connective tissue was present (Figure 2A). In the exocrine pancreas of WD-fed mice, many necrotic cells were seen (Figures 2C, D). In most of the acinar cells, the cytoplasm contained numerous vacuoles (Figures 2C, D). Using light microscopy and transmission electron microscopy, extracellular accumulations of lipids in the connective tissue enveloping the pancreas were never observed in CD-fed mice but were present occasionally in WD-fed mice. The structure of the analyzed tissue was comparable in all individuals within both tested groups of animals.

### Transmission electron microscopy (TEM)

#### Control diet-fed mice

**FIGURE 3:**
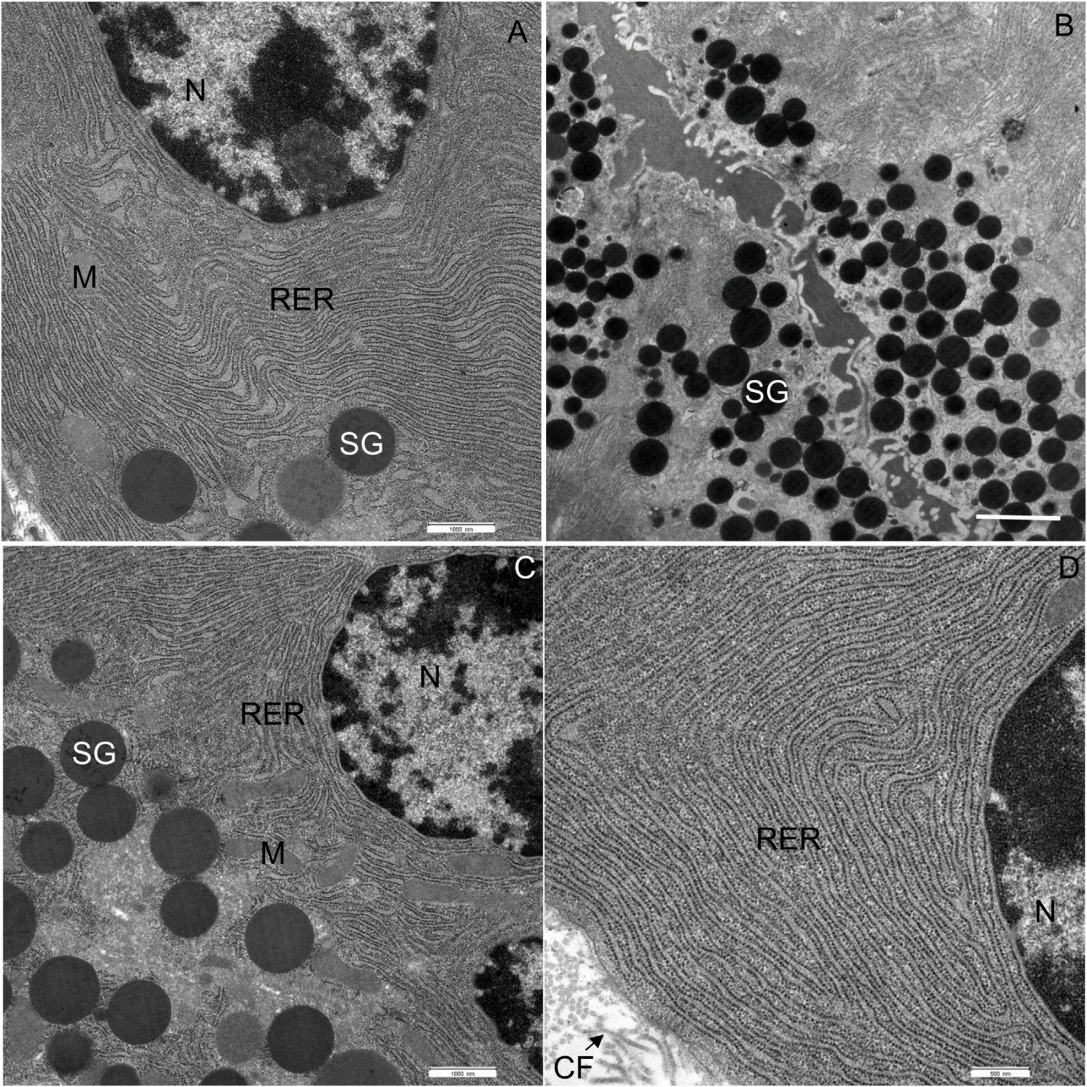
Ultrathin section of the pancreas of control mice. **(A)** The nucleus (N), located centrally, is surrounded by numerous cisternae of rough endoplasmic reticulum (RER) and secretory granules (SG). **(B)** Numerous secretory granules (SG) are located in the apical part of the cell. **(C,D)** Rough endoplasmic reticulum (RER) showing typical structure with flattened cisternae. Beneath the acinar cell, some collagen fibres (CF) are seen. M, mitochondrium. Scale bars: **(A)** 1 μm; **(B)** 2 μm; **(C)** 1 μm; **(D)** 500 nm.

In CD-fed mice, the exocrine cells were characterized by an abundant rough endoplasmic reticulum (RER) and many electron-dense secretory granules (Figures 3A–D). The cisternae of the RER (Figures 3A, C, D) were tightly packed in different parts of the cell. Secretory granules were present in the perinuclear (Figures 3A, C) and predominantly the apical cytoplasm (Figure 3B). The acinar cells also contained typical elongated mitochondria with well-defined cristae and an electron-dense matrix. The mitochondria were present in all parts of the cell. The nucleus was round and located centrally or basally. No necrotic cells were found in any of the tissue samples of CD-fed mice. None of the analyzed acinar cells displayed any aberrant ultrastructural features.

#### Western diet-fed mice

**FIGURE 4:**
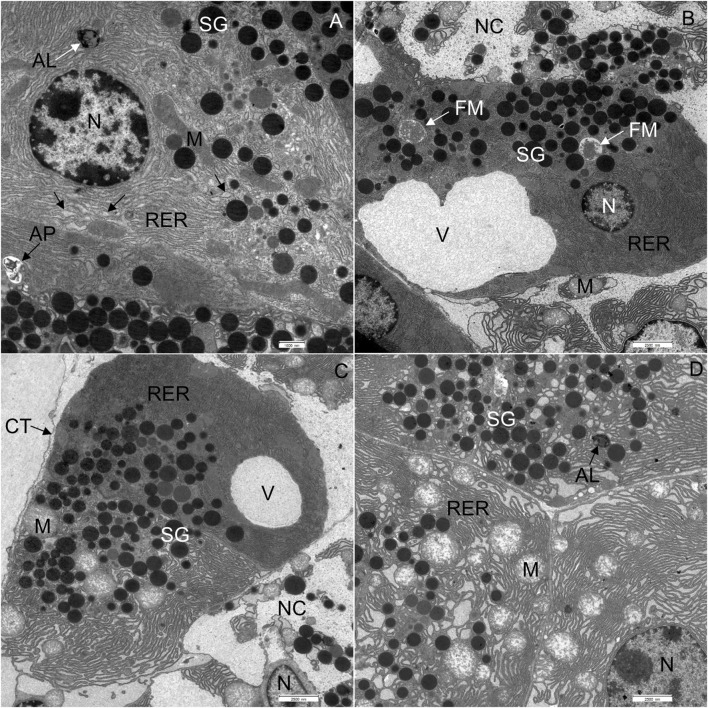
Ultrathin section of the pancreas of mice fed a Western diet. **(A)** Some cisternae of the rough endoplasmic reticulum (RER) had a wider lumen in comparison to those in control mice. **(B)** Acinar cell containing one larger vacuole. **(C)** One acinar cell with larger vacuole and the other with changed structure of RER and mitochondria (M). **(D)** All acinar cells with changed structure of RER and mitochondria (M). AL, autolysosome; AP, autophagosome; CT, connective tissue; N, nucleus; NC, necrotic cell; SG, secretory granulum; V, vacuole. Scale bars: **(A)** 1 μm; **(B–D)** 2.5 µm.

**FIGURE 5:**
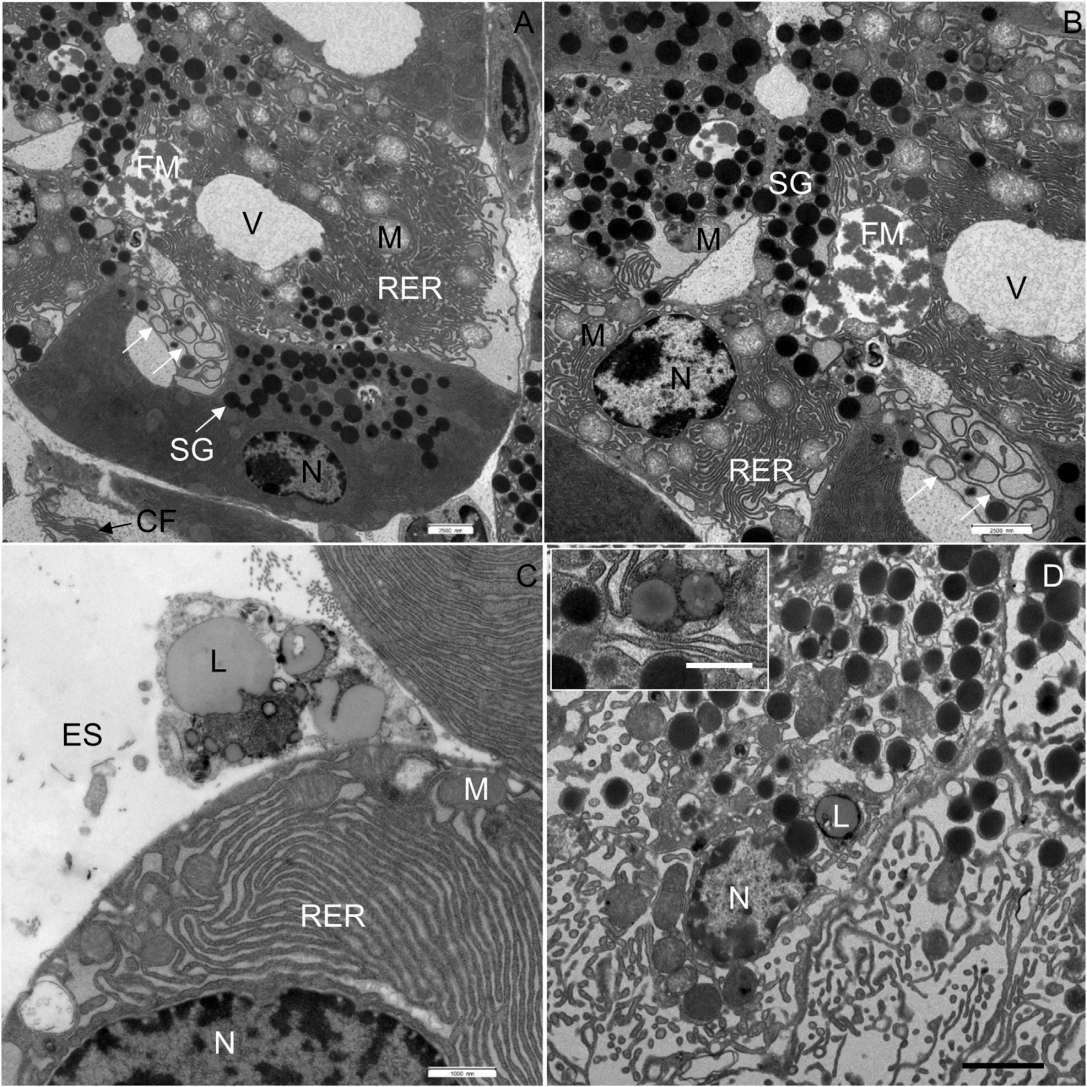
Ultrathin section of the pancreas of mice fed a Western diet. **(A)** In the acinar cell in the middle of the micrograph, two vacuoles (V) are seen, one with flocculent material (FM). Disorganized membranes of the RER are indicated by white arrows. **(B)** Higher magnification of image **(A)**; white arrows show the swirls of degenerated RER. **(C)** Lipid droplets were found in the extracellular space (ES). **(D)** Additionally, lipids (L) had accumulated in the acinar cells. CF, collagen fibres; M, mitochondrium; N, nucleus; RER, rough endoplasmic reticulum; SG, secretory granulum; V, vacuole. Scale bars: **(A,B)** 2.5 μm; **(C)** 1 μm; **(D)** 2 µm (Inset: 1 µm).

In WD-fed mice, the ultrastructural characteristics (Figure 4) of the acinar cells differed from those of CD-fed mice (Figure 3, and these were observed consistently in all analyzed pancreata. The acinar cells of WD-fed mice contained as abundant an RER as the acinar cells in control mice, but the predominant part of the RER (Figures 4A, D, 5A–D) was changed in numerous acinar cells; its cisternae became expanded and consequently, the lumen of the cisternae appeared wider. We further quantified this observation by detecting RER cisternae on individual TEM images and calculated the percentage of cross-sectional area populated by the RER cisternae (% RER area, Figure 7). While in the CD-fed mice the RER cisternae occupied an area of about 10% (12.4 ± 2.0%), this was almost four-fold larger (39.4 ± 2.2%) in the WD-fed mice.

**FIGURE 6:**
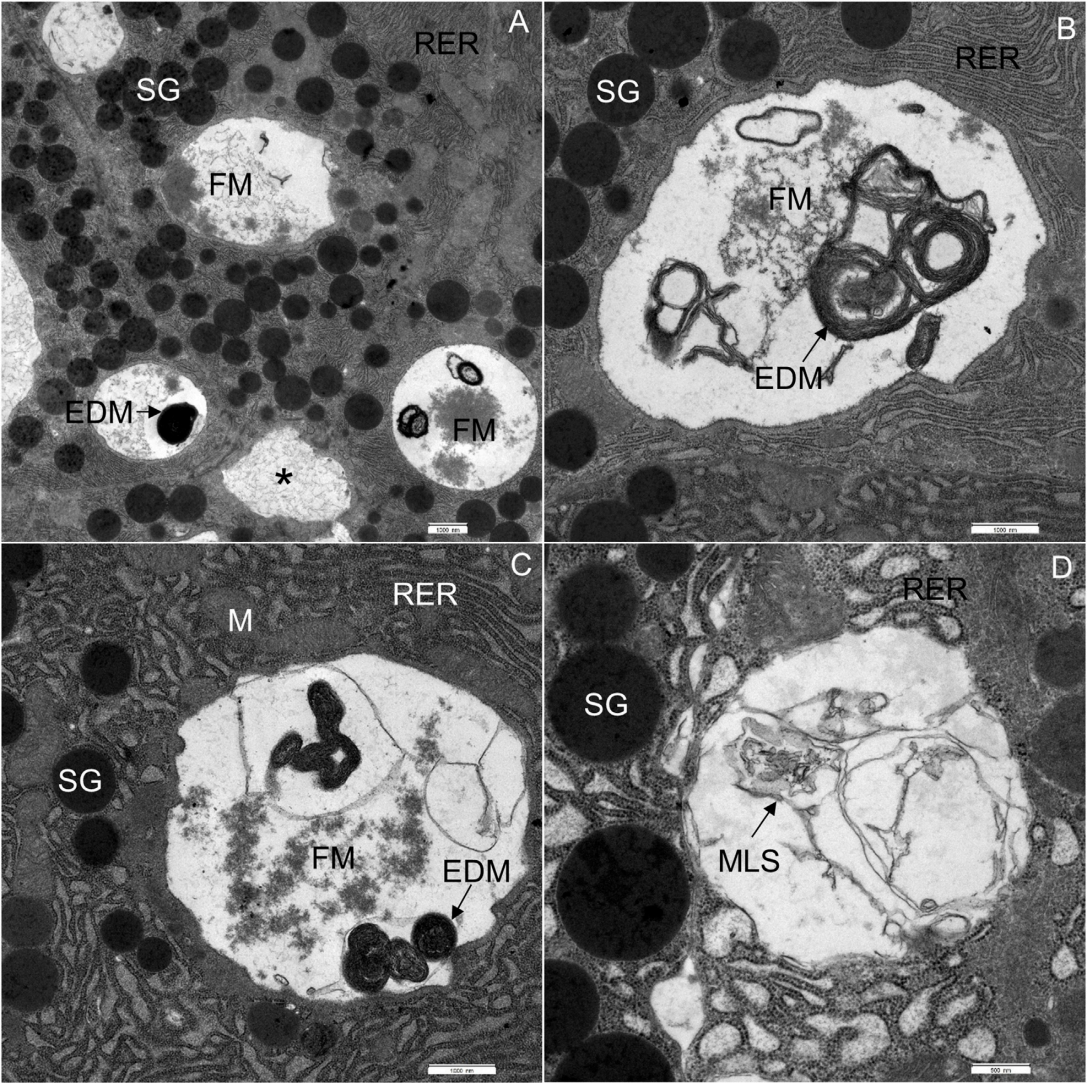
Ultrathin section of the pancreas of mice fed a Western diet. **(A)** Acinar cells, containing multiple vacuoles: vacuoles with homogeneous material (asterisk), flocculent material (FM) and electron-dense material (EDM). **(B)** Acinar cell, containing flocculent material (FM) and electron-dense material (EDM). **(C)** Acinar cell, containing flocculent material (FM), electron-dense material (EDM) and some membrane-like structures. **(D)** Acinar cell, containing some membrane-like structures (MLS). M, mitochondrium; RER, rough endoplasmic reticulum; SG, secretory granulum. Scale bars: **(A–C)** 1 μm; **(D)** 500 nm.

**FIGURE 7:**
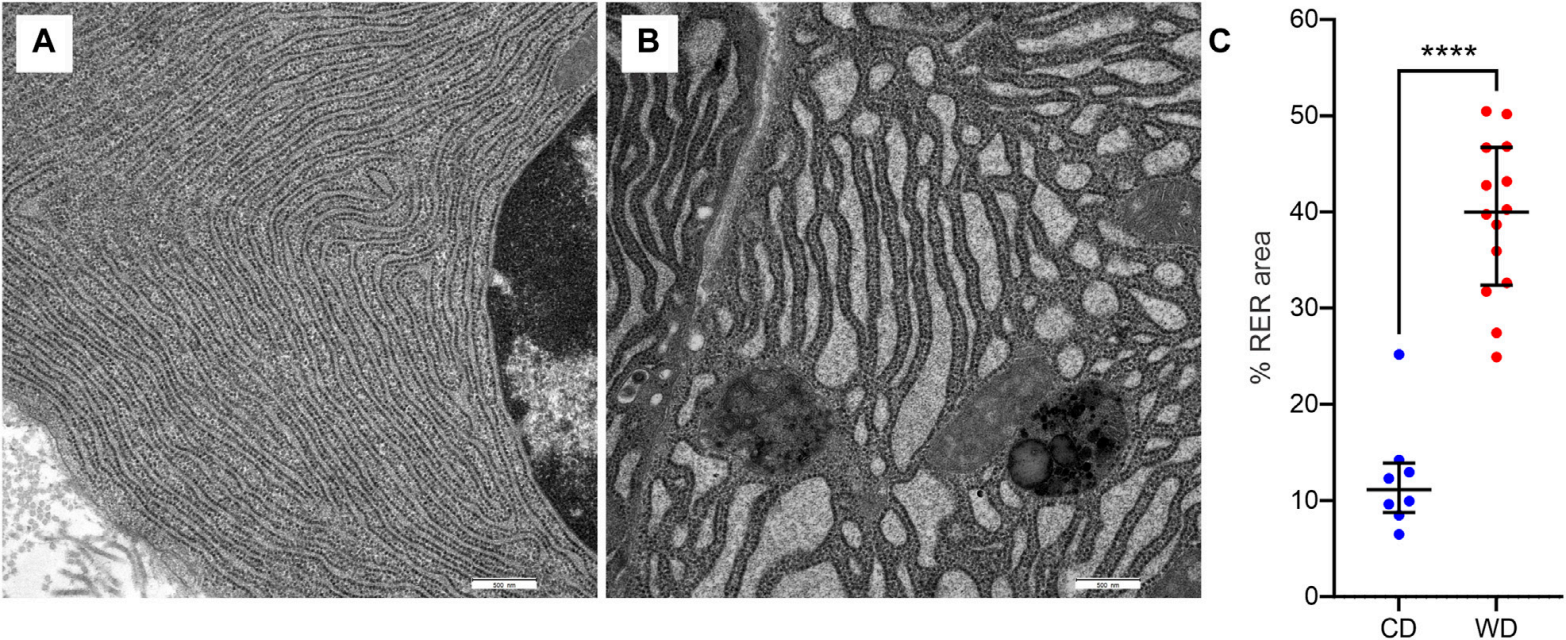
WD-induced changes in rough endoplasmic reticulum (RER) morphology. **(A,B)** Representative images of RER from CD-fed **(A)** and WD-fed **(B)** animals. **(C)** Quantitative analysis of RER morphology expressed as percentage of cross-sectional area populated by RER cisternae in CD-fed (blue) and WD-fed (red) animals. Data pooled from 8 to 12 TEM images from CD- and WD-fed animals, respectively, and expressed as individual data points in addition to mean and SEM. ****p < 0,0001 (Student’s *t*-test).

**FIGURE 8:**
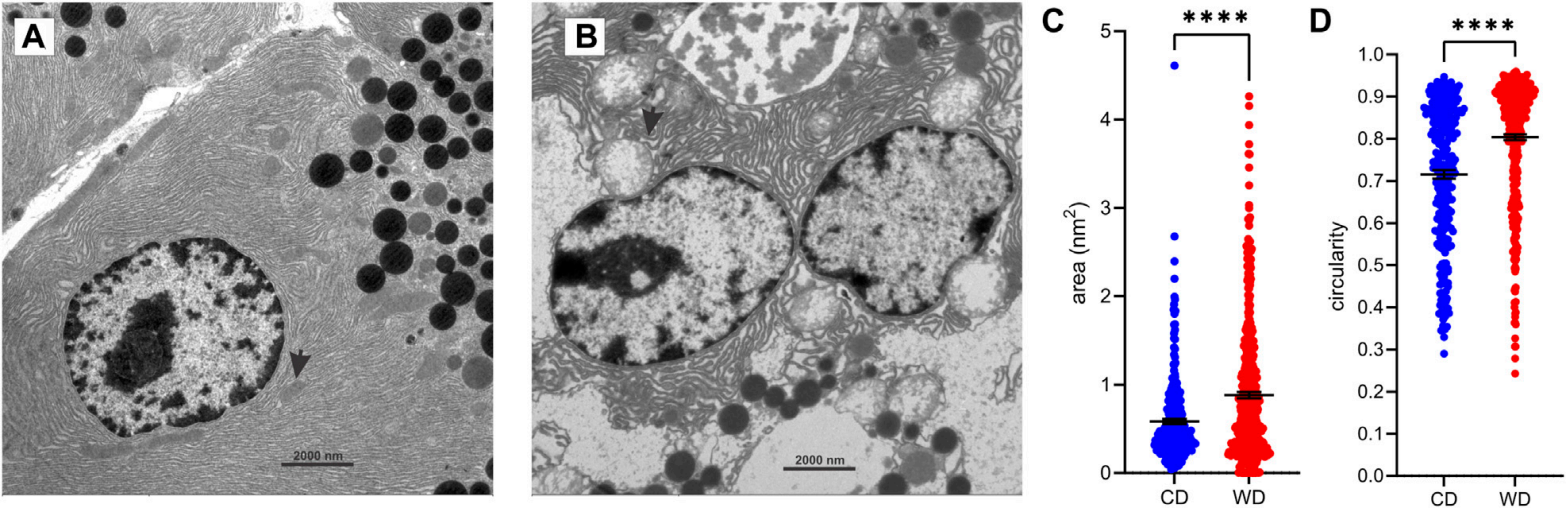
WD-induced changes in mitochondrial morphology. **(A,B)** Representative ultrastructural images of acinar cells from CD-fed **(A)** and WD-fed **(B)** animals. Arrows point to mitochondria. **(C,D)** Quantitative analysis of mitochondria. Cross-sectional area **(C)** and circularity **(D)** in CD-fed (blue) and WD-fed (red) animals. Data pooled from 278 to 449 mitochondria from 15 to 15 TEM images from CD- and WD-fed animals, respectively, and expressed as mean and SEM. ****p < 0,0001 (Student’s *t*-test).

Furthermore, extensive vacuoles were observed in the cytoplasm (Figures 4B, C), as well as individual autophagosomes and autolysosomes (Figure 4A). The mitochondria were larger and mostly rounded, with less-well-defined cristae and a more electron-lucent matrix (Figures 4D, 5A). We quantified the WD-induced mitochondrial changes in Figure 8, demonstrating an about 50% increase in their size (0.58 ± 0.03 in CD-fed and 0.88 ± 0.04 nm^2^ in WD-fed animals) and a tendency toward a more circular shape (circularity 0.72 ± 0.01 and 0.80 ± 0.01 in CD- and WD-fed animals, respectively).

**FIGURE 9:**
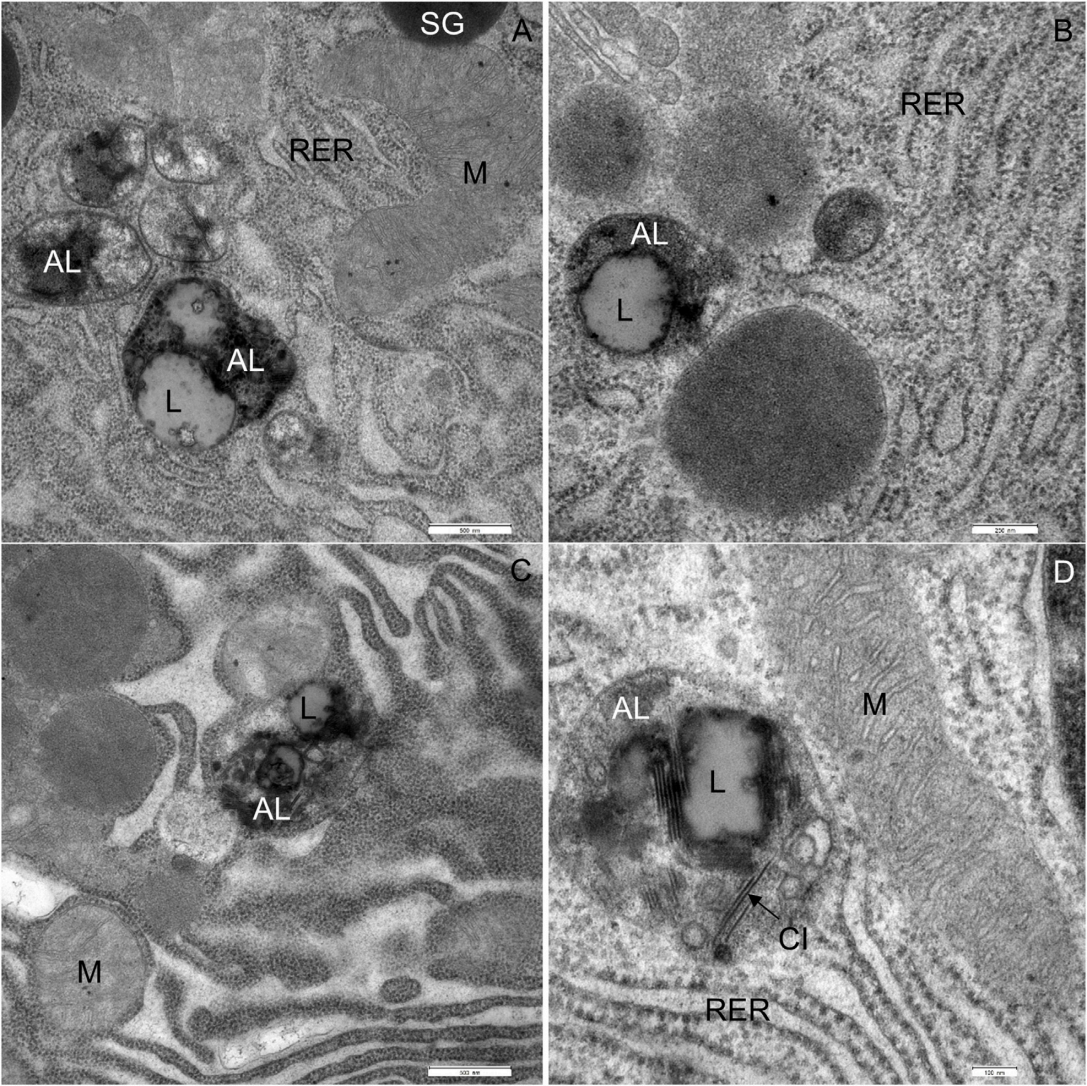
Ultrathin section of the pancreas of mice fed a Western diet showing autophagic structures. **(A–D)** The autolysosomes (AL) are composed of the material of varying electron density and lipids (L). CI, crystalline-like inclusions M, mitochondrium; RER, rough endoplasmic material; SG, secretory granulum. Scale bars: **(A,C)** 500 nm; **(B)** 250 nm; **(D)** 100 nm.

The most significant characteristic was the vacuolization of the cytoplasm. Many acinar cells contained vacuoles with different structural characteristics: 1) vacuoles containing a homogenous, electron-lucent material (Figures 4B, C); 2) vacuoles with a flocculent material (Figures 4B, 5A, B); 3) vacuoles containing a flocculent and an electron-dense material (Figures 6A–C); and 4) vacuoles containing a membrane-like material (Figure 6D). Additionally, some membranes of the RER were disorganized and formed swirls in the cytoplasm (Figures 5A, B). In the exocrine tissue, lipid droplets were present both extracellularly (Figure 5C) and intracellularly. In the latter case, they appeared in the form of both individual lipid droplets in the cytoplasm (Figure 5D) and lipid inclusions in the autophagic structures (e.g., in Figures 9A–D). Many acinar cells contained autophagic structures: autophagosomes, autolysosomes, and residual bodies. The most predominant autophagic structures were the autolysosomes. These were composed of a material of varying electron density (Figures 9A–D). Numerous autolysosomes additionally contained lipids, membrane fragments, and crystalline-like inclusions (Figures 9C, D).

**FIGURE 10:**
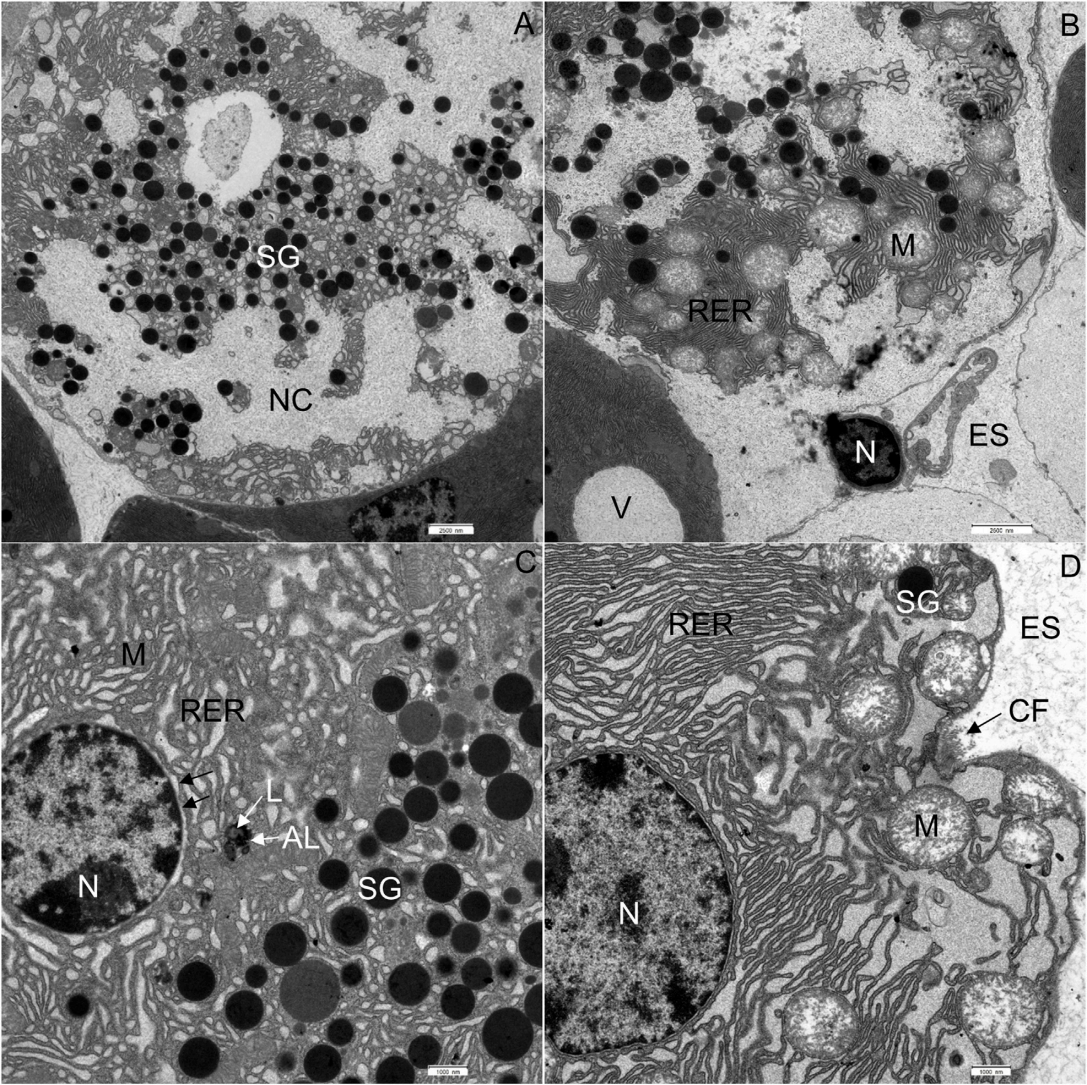
Ultrathin section of the pancreas of mice fed a Western diet. **(A)** Necrotic cell with disorganized rough endoplasmic reticulum (RER) and mitochondria (M). **(B)** The nucleus (N) of the necrotic cell has been extruded into the extracellular space (ES). The RER and mitochondria are disorganized. **(C)** The outer nuclear membrane (indicated by black arrows) is detached from the nucleus. The RER is disorganized. The autolysosome (AL) is composed of electron dense material and lipids (L). **(D)** Acinar cell with disorganized RER and round mitochondria (M). The cristae could not be recognized. CF, collagen fibres; ES, extracellular space; N, nucleus; NC, necrotic cell; SG, secretory granulum; V, vacuole. Scale bars: **(A,B)** 2.5 μm; **(C,D)** 1 µm.

Finally, the exocrine tissue was characterized by numerous necrotic cells. In the tissue of WD-fed mice, on average, 7 necrotic cells were seen on the surface of 0.12 mm^2^. The cytoplasm of necrotic cells underwent vacuolization and became electron-lucent (Figures 10A–D). The cell membranes were disrupted, while RER and mitochondria were disorganized (Figures 10A–D). Mitochondria were round, and the inner membrane was not organized in typical cristae. The outer nuclear membrane was detached from the nucleus (Figure 10C). When the necrosis was in its final stage, the apical plasma membrane broke, and the cytoplasm, the nucleus, and other cell compounds were discharged into the extracellular space (Figure 10B).

## Discussion

Numerous studies have described the presence of exocrine insufficiency in type 1 and type 2 diabetes mellitus (Hardt et al., 2003), with changes ranging from reduced enzyme secretion and cell atrophy, to reduced pancreatic mass (Lankisch et al., 1982; Campbell-Thompson et al., 2015; Foster et al., 2020; Overton and Mastracci, 2022). However, a detailed qualitative description of the ultrastructural changes in the pancreatic acinar cells, especially in the setting of type 2 diabetes, is largely missing (de Boer et al., 2020). We therefore resorted to a mouse model in which 8 weeks of WD feeding results in obesity with an insufficiently compensated type 2 diabetes mellitus (Paschen et al., 2019). We systematically analyzed structural and ultrastructural changes in acinar cells of WD-fed mice and compared them qualitatively and quantitatively with normal structural and ultrastructural features of acinar cells in CD-fed mice.

Using light microscopy, we observed that the WD intervention caused changes in the acinar cells, highlighted by intense vacuolization of the cytoplasm and the presence of many necrotic cells. To substantiate this into more detail, we analyzed the ultrastructural properties of acinar cells using TEM. Using this approach, we corroborated that the cytoplasm of numerous acinar cells showed intense vacuolization in WD-fed mice. The extent of vacuolization was comparable to or even more severe than in acute pancreatitis (Kui et al., 2015; Klöppel, 2018). In previous studies, the appearance of vacuoles in the cytoplasm was ascribed to long-lasting stimulation of pancreatic acinar cells (Tapp, 1970). By analogy, in our case the vacuoles may be due to increased levels of acinar secretion precipitated by the high amounts of ingested lipids (Go, 1993; Keller and Layer, 2005; Birk et al., 2014). It should be pointed out, however, that the level and activity of amylase in the pancreas have not changed in our study. Additionally, other studies have reported reduced levels of specific pancreatic enzymes in HFD-induced obesity in C57BL/6J mice after 12 weeks, while enzyme levels increased transiently at the start of the diet (Birk et al., 2014). Abnormalities in enzyme secretion were also reported in other diabetic animal models (Bendayan and Grégoire, 1987), in patients with type 1 diabetes (Lankisch et al., 1982), and type 2 diabetes. In the latter, patients with a more progressive form of the disease had a higher prevalence of exocrine pancreatic deficiency, defined as a state in which pancreatic enzyme activity is below the threshold required to maintain normal digestion (Zhang et al., 2022). We hypothesize that the duration of dietary intervention in our case was insufficient to induce full-blown exocrine pancreatic dysfunction that would lead to reduced enzyme secretion; instead, it resulted in a compensatory increase in enzyme secretion and extensive vacuolization. Further studies are needed in WD-fed mice over a longer period and mice on caloric restriction to attempt a reversal of the observed changes to clarify the mechanism behind the changes described above.

Another striking feature of the WD intervention was the structural transformation of the mitochondria, many of which finally degenerated. Structurally altered mitochondria were characterized by their oval-to-round form and barely recognizable cristae. The inner membrane of numerous mitochondria was not organized in characteristic cristae. Additionally, in the matrix of mitochondria, only fragments of the inner mitochondrial membrane were observed. Similar changes in mitochondria have been observed in streptozotocin (STZ)-induced diabetic rats, where swollen mitochondria with loss of cristae and degenerative changes have been described (Yoon et al., 2011). In this vein, in the alloxan-induced diabetes rat model analogous ultrastructural changes also appeared in acinar cell mitochondria (Titova et al., 2020). Finally, changes in mitochondrial structure were reported as one of the main features of necrotic cell death (Niemann and Rohrbach, 2016), suggesting necrotic processes following WD intervention.

Moreover, we observed various structural abnormalities in the organization of the RER in WD-fed mice. Enlarged RER tubules arranged in parallel with each other and containing electron-lucent secretory material have been shown in STZ-induced diabetes models in rats (Yoon et al., 2011) and dogs (Yasuda et al., 1982). In our case, WD intervention resulted in similarly enlarged cristae of the RER. We hypothesize that RER alterations could be explained by adaptive reactions to the increased demand for protein synthesis caused by the WD, eventually resulting in ER stress and degeneration (Sah et al., 2014; Yatchenko et al., 2019).

WD-induced obesity and T2DM were also associated with fat accumulation in the pancreas and development of NAFPD with features like those shown in humans (Sah et al., 2014; Della Corte et al., 2015). WD intervention resulted in many lipid droplets in the cytoplasm and inclusions in the autophagic structures in the acinar cells. By analogy with NAFLD, it can be speculated that at least some part of the excess energy accumulates in the cytoplasm of acinar cells in the form of triacylglycerides (Petrov and Taylor, 2022). Lipid accumulation has been shown before in various diabetic rodent models (Lee et al., 2010). In Zucker diabetic fatty rats that were fed a HFD from 6 weeks of age, lipid droplets were found in the acinar cells at 12, 18, and 24 weeks of age (Matsuda et al., 2014), while HFD-fed Wistar rats showed adipose infiltration in intra- and extra-lobular locations, which decreased after bariatric surgery (Rebours et al., 2018). In mice, similar morphological changes, e.g., lipid accumulation in the cytoplasm and changed structure of some organelles, were reported in the renal cells of HFD-induced obese individuals (Deji et al., 2009; Declèves et al., 2014). A similar effect was also shown in dogs where diabetes was induced by STZ (Yasuda et al., 1982).

Furthermore, in two rat models of HFD fibrosis developed in the pancreas (Matsuda et al., 2014; Rebours et al., 2018). At 12 weeks of age, fibrosis was rarely seen in the pancreatic tissue of Zucker rats, but it was significant at 24 weeks of age (Matsuda et al., 2014). In our study, in mice aged 12 weeks and analyzed at 20 weeks of age, we could find no signs of fibrosis, and further studies are needed to assess whether it would develop after longer exposure to WD.

In all the examined WD-fed mice, together with cell degeneration, the process of autophagy was striking in numerous acinar cells. In previous studies, autophagic structures were reported in HFD-fed mice (Rouse et al., 2014). Autophagy is an adaptation process of cells to stress conditions (Mizushima, 2007; Mizushima et al., 2008; Klionsky et al., 2021). In cells sufficiently supplied with nutrients to maintain their normal functioning, autophagy is suppressed. Otherwise, it is induced by a range of stress factors, e.g., starvation (Munafó and Colombo, 2001; Mizushima, 2004; Romanelli et al., 2014; Wilczek et al., 2014), hormone stimulation (Franzetti et al., 2012), microsporidian infection (Böni‐Schnetzler et al., 2008), chemical substances (Wilczek et al., 2014), and disease (Mizushima, 2007). Autophagy is a cell process responsible for the degeneration of cytoplasmic components and can help maintain homeostasis (Mizushima et al., 2008). Selective autophagy, where specific cell organelles and compounds are degraded, e.g., the mitochondria (mitophagy), endoplasmic reticulum (reticulophagy), or lipids (lipophagy) (Liu and Czaja, 2013; Klionsky et al., 2021), was not detected in our case; instead, WD intervention resulted in non-selective autophagy, with different types of organelles and compounds being observed in autophagosomes, suggesting that WD induces a more general compensatory autophagic response in acinar cells. Autophagy can either protect against or facilitate cell death (Levine and Klionsky, 2004; Klionsky et al., 2021). In WD-fed mice, various cell compounds and degenerated organelles were enclosed in autophagosomes. After fusion of the autophagosomes with the lysosomes, autolysosomes are formed, and their components are disintegrated (Mizushima, 2007; Klionsky et al., 2021). We postulate that in WD-fed mice, autophagy becomes activated to reduce the influence of abundant nutrients. In the examined acinar cells, numerous autolysosomes contained lipid inclusions, and it seems that autophagy is an important, albeit obviously insufficient, pro-survival process in exocrine cells under stress conditions caused by ER stress, mitochondrial dysfunction, and the abundance of lipids and other energy-rich nutrients in WD. In general, once macromolecules become degraded in autolysosomes, monomers, e.g., amino acids and fatty acids, are released to the cytosol for reuse (Klionsky et al., 2021).

If the cytoplasm is filled with numerous autophagic structures, these can be harmful to the cell, and consequently, cell death can be activated (Levine and Klionsky, 2004). The consequences of stress involve changes in polypeptide synthesis, denaturation of proteins, DNA damage, disorders in intracellular respiration, and disintegration of cellular structures (Mizushima et al., 2008; Klionsky et al., 2021). When a cell can no longer stand the extent of the damage, it carries out necrotic and/or apoptotic changes and dies (Klionsky et al., 2021). In the exocrine cells of WD-fed mice, the process of apoptosis was not observed; on the contrary, the process of necrosis was significant. Necrosis or necrotic cell death is defined as incidental, passive cell death, initiated by disruptive external factors (e.g., physical, chemical or/and biotic factors) that cause a number of morphological changes, loss of osmotic pressure and swelling of cells (McCall, 2010). According to our results, it seems that WD is a major disruptive external factor, with necrosis predominating over apoptosis, but further quantitative studies are needed to define this in more detail. Finally, since tissue and plasma levels and activity of amylase are often used clinically to track the development of pancreatic dysfunction, it is worth pointing out that in our case, all of the above structural changes were observed without any detectable changes in amylase, emphasizing the importance of using other diagnostic approaches for early detection of pancreatic dysfunction (Lateraza et al., 2013; Nøjgaard et al., 2013; Nakajima, 2016).

Regarding the translational relevance of our findings in the mouse model for the human acinar cells during development of type 2 diabetes, to the best of our knowledge there is a lack of studies systematically comparing the ultrastructure of exocrine tissue in human donors with and without T2DM, which is mostly due to the difficult access to human tissue (Gukovskaya et al., 2019; Atkinson et al., 2020; Odugbesan et al., 2024). However, the spectrum of ultrastructural changes observed in our case is comparable with the disordering of acinar cell ER, endolysosomal system, mitochondria, and autophagy observed in tissue sections from patients with pancreatitis (Helin et al., 1980; Fortunato et al., 2009; Mareninova et al., 2009; Biczo et al., 2018), as well as in isolated human acinar cells (Gukovskaya et al., 2016; Lugea et al., 2017; Gukovskaya et al., 2019) and acinar cells in tissue slices (Xu et al., 2017; Dolai et al., 2018) subjected to pancreatitis insults *in vitro.* Moreover, our findings of both intra- and extracellular lipid material together with ultrastructural changes suggesting low grade pancreatitis in the setting of T2DM are consistent with similar findings in human patients with T2DM and pancreatitis (Petroff et al., 2021; Caldart et al., 2023), as well as with the recently proposed hypothesis that these pancreatic diseases may originate from intrapancreatic fat or start with NAFPD (Alempijevic et al., 2017; Petrov, 2023; Pagkali et al., 2024).

## Conclusion

The development of new modalities for detection of structural changes in the pancreas in the clinical environment has revolutionized our understanding of the role of changes such as intrapancreatic fat deposition in the development of acute and chronic pancreatitis, diabetes mellitus, and pancreatic cancer. Diffuse intrapancreatic fat deposition or NAFPD is much more frequent than the aforementioned diseases combined, and it represents an early morphological substrate of pancreatic exo- and endocrine dysfunction. It also seems to be reversible, with concomitant decreases in the risk for advanced pancreatic disease (Taylor et al., 2018; Petrov and Taylor, 2022). However, studies in humans are inherently limited in terms of temporal and spatial resolution, as well as sample size and characteristics, and thus translationally relevant studies in animal models are essential to detect the earliest cellular defects in disease pathogenesis, as well as to determine the point of no return, when such changes become irreversible and clinical improvements less probable (Taylor et al., 2018). In our present study, we have established a new preclinical methodological pipeline for studying changes in acinar cell structure during the development of diet-induced obesity and T2DM and found that early during the development of obesity and progression from compensated to decompensated diabetes (Paschen et al., 2019), acinar cells already show striking ultrastructural signs of functional adaptation to increased workload and dysfunction affecting intracellular organelles in a rather non-selective manner and before changes in tissue or plasma levels and activity of amylase become apparent. Our approach is methodologically compatible with functional studies of acinar cells (Marolt et al., 2022), with morphological and functional studies of other cell types in the exocrine and endocrine parts of the pancreas (Marciniak et al., 2014; Stožer et al., 2021), as well as with more quantitative image-analysis approaches (Klemen et al., 2022). These should be combined in the future to obtain more answers regarding the relationship between structural and functional changes in endocrine, ductal, and acinar cells during disease progression, regarding the relationship between the various cell types, as well as regarding the reversibility of these changes. Future studies shall also be directed towards investigating sex differences by including female mice at specific stages of estrous cycle, as well as towards tracking the development of ultrastructural and functional changes in mice exposed to WD for a longer period of time, which could help us better understand the extent of structural pathological changes required for overt dysfunction detectable at the enzyme activity level, as well as their temporal relationship with T2DM development.

## Data Availability

The original contributions presented in the study are included in the article/Supplementary Material, further inquiries can be directed to the corresponding authors.

## Supplementary material

Supplementary Figure S1 ipGTT before the start of the diet intervention. CD-fed animals depicted in blue and WD-fed animals fed in red. No statistical differences were detected (2-ANOVA followed by Bonferroni’s post hoc test).
