# Supplementary figures and images for: Western diet-induced ultrastructural changes in mouse pancreatic acinar cells
Source: Front Cell Dev Biol. 2024 Mar 14;12:1380564. doi: 10.3389/fcell.2024.1380564 (PMC10972872; doi:10.3389/fcell.2024.1380564)

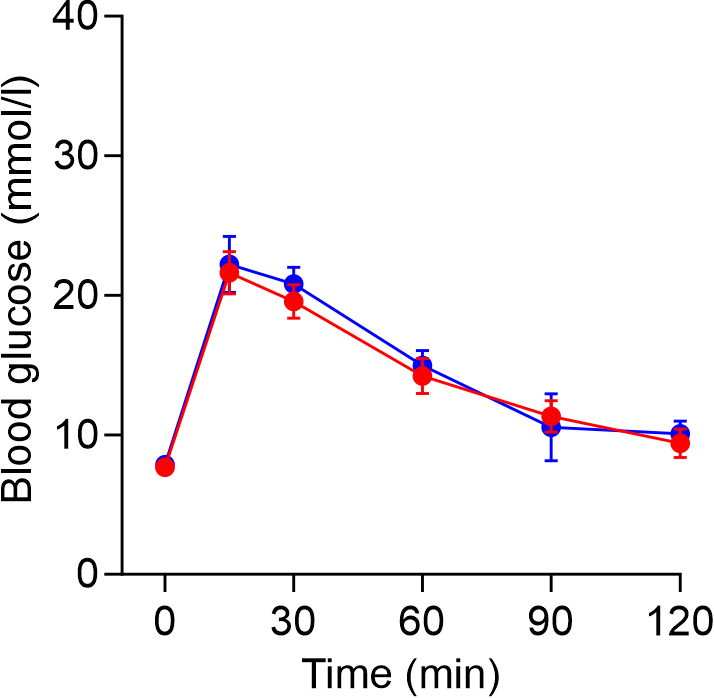

Supplement: Supplementary file 1 [file Image1.tif]
